# Measuring Patient-Reported Outcomes Following Traumatic Craniomaxillofacial Injuries: Development of the AO CMF Injury Symptom Battery

**DOI:** 10.3390/jcm13237156

**Published:** 2024-11-26

**Authors:** Sally E. Jensen, Nan E. Rothrock, Leilani Lacson-Soltysiak, Alexis Olsson, Edward Ellis

**Affiliations:** 1 Outcomes Research Network, Research Institute, Endeavor Health (formerly NorthShore University HealthSystem), 1001 University Place, Evanston, IL 60201, USA; 2 Department of Medical Social Sciences, Northwestern University Feinberg School of Medicine, 625 N. Michigan Ave. 27th Floor, Chicago, IL 60611, USA; n-rothrock@northwestern.edu; 3 Equal Hope, 300 S. Ashland Ave. #202, Chicago, IL 60607, USA; leilani_l_lacson@rush.edu; 4 Department of Clinical Otolaryngology-Oral & Maxillofacial Surgery, Northwestern University Feinberg School of Medicine, NMH/Galter Room 12-100, 210 E. Huron, Chicago, IL 60611, USA; aolsson@nm.org; 5 Department of Oral and Maxillofacial Surgery, University of Texas Health Science Center at San Antonio, 8210 Floyd Curl Drive, MC 8124, San Antonio, TX 78229, USA; ellise3@uthscsa.edu

**Keywords:** traumatic craniomaxillofacial injury, patient-reported outcome, symptom, measure development

## Abstract

**Background/Objectives:** Traumatic craniomaxillofacial (CMF) injuries are associated with various symptoms/concerns that affect patients’ quality of life. The assessment of outcomes from the patient perspective has been limited by the absence of patient-reported outcome (PRO) measures tailored to this patient population. To address this need, we employed a mixed methods, multi-step process to first identify the most important symptoms/concerns and then use this information to construct a PRO symptom battery. **Methods:** CMF clinicians and patients who had sustained traumatic CMF injuries participated in semi-structured interviews to elicit the symptoms/concerns considered the most important. The data were analyzed using an iterative coding procedure and symptom/concern frequency was tabulated. The findings were used to develop a conceptual model of the most important symptoms to include in a PRO battery. Existing items were modified as needed and new items were drafted to ensure adequate coverage of the symptoms. **Results:** The resulting AO CMF Injury Symptom Battery includes four modules specific to the injury site (oral, ocular, nasopharyngeal, ear) and five universal modules (pain/sensation, cognitive, cosmetic, psychosocial, and injury impact). **Conclusions:** The AO CMF Injury Symptom Battery offers promise for assessing symptoms only patients can report on in clinical research and practice. Ongoing research will examine the battery’s psychometric properties.

## 1. Introduction

Traumatic craniomaxillofacial (CMF) injuries affect many aspects of health-related quality of life. Depending upon the injury location, severity, and treatment, patients may experience pain, changes to function, and changes to satisfaction with physical appearance, among other impacted areas [1,2,3,4].

Patient-reported outcome measures (PROs) are useful in assessing symptoms that require the patient’s perspective. PROs have been defined in various ways, although the core principle of PROs is, as the name implies, the incorporation of the patient’s perspective when assessing outcomes: “…any report of the status of a patient’s health condition, health behavior, or experience with health care that comes directly from the patient, without interpretation of the patient’s response by a clinician or anyone else [5]”. The PRO findings reflect the patient’s voice and their perception of their unique experience. PROs have been applied in both clinical research in practice to explore a wide variety of domains, including (but not limited to) quality of life, health status, health behaviors, healthcare experience, symptoms/concerns, and physical/social/emotional functioning. PRO measures can include both generic (applied across conditions) assessments, as well as condition-, symptom-, or treatment-specific assessments. Their use has been widely incorporated into clinical research (e.g., clinical trials), clinical practice (e.g., symptom monitoring, shared decision-making), as well institutional healthcare initiatives (e.g., quality improvement, performance measurement). PROs can be administered using a variety of different methods (e.g., self-report, proxy report), modalities (e.g., paper/pencil, electronic), and settings (e.g., clinic, home).

Although PROs’ use in clinical practice has dramatically increased, they have been less widely used among CMF surgeons. In a study examining the knowledge and use of PROs in a large international sample of trauma, spine, and CMF surgeons, Joeris and colleagues found that compared to the other disciplines sampled, CMF surgeons reported the least familiarity and use of PROs in clinical practice [6]. The authors posited that CMF surgeons’ low familiarity with PROs may reflect the paucity of well-constructed CMF PROs [6]. To date, PROs have not been as widely developed to measure outcomes specifically in CMF injuries compared to other conditions. Although a wide variety of PROs is available for CMF conditions such as head and neck cancer [7,8], dental conditions [9,10], temporomandibular joint surgery [11], and congenital conditions [12], at this time, no standard PRO measure exists in traumatic CMF injury research or clinical practice. In a meta-analysis that, in part, examined PROs in individuals with soft tissue facial injury, Wong and colleagues concluded that there are limited data on the use of PROs in facial injury and that previous research in this area predominantly relied upon the use of generic measures not developed specifically for this population [13]. The absence of a measure to examine PROs in individuals with traumatic CMF injuries limits the ability to know the full impact of these injuries and their treatments. It also limits clinical research to determine the effectiveness of interventions for individuals with traumatic CMF injuries.

In response to the need for PROs to assess outcomes in individuals with traumatic CMF injuries, this project developed a battery of PRO measures to evaluate outcomes after trauma-related CMF injuries in clinical research and practice. The goal of this project was to utilize gold standard measure development methods incorporating both provider and patient input in order to fill the current gap in available PROs specific to this population. The specific aims of the proposed project included identifying the CMF symptoms perceived as most important by patients and clinical experts to monitor following traumatic CMF injuries and constructing a traumatic CMF injury symptom battery based on expert and patient input.

## 2. Materials and Methods

### 2.1. Design

**Figure 1:**
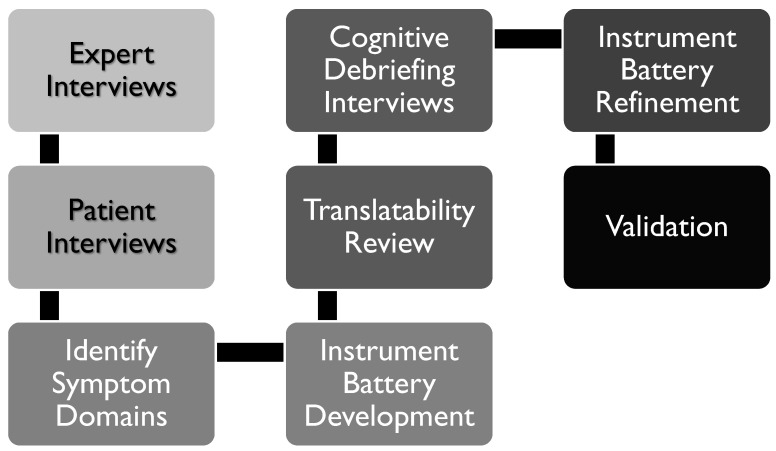
Multi-step battery development process.

The development of the AO CMF Injury Symptom Battery was a multi-step process (Figure 1). Prior to the start of the study, a literature review of PRO assessment in a wide variety of CMF patient populations was conducted, revealing a notable absence of PROs developed for traumatic CMF injuries. Most studies identified in this literature review either utilized generic non-condition-specific PROs, involved pediatric populations, or were conducted in non-traumatic CMF populations (e.g., orofacial/dental, cancer). The findings from the literature highlighted the importance of a PRO tool targeted specifically at traumatic CMF injury. The literature review was also informative in the development of expert and patient interview guides, as well as identifying candidate items for inclusion in the symptom batter.

As the next step in this phase of the study, semi-structured concept elicitation interviews were conducted with clinical experts and patients to identify the most important symptoms to measure when assessing outcomes following traumatic CMF injuries. The findings from these interviews informed the development of a conceptual model, which contained the symptom domains most important to include in the symptom battery. Based upon the conceptual model, existing relevant items from validated measures were reviewed and included when possible, and new items were generated for content areas not covered by existing items. Newly generated items were reviewed for translatability. Cognitive debriefing interviews were conducted with patients to assess the appropriateness of inclusion in the final symptom battery and comprehension. Items were modified as needed and organized into specific symptom domains. This study was determined by the Northwestern University Institutional Review Board and the University of Texas Health Science Center at San Antonio Institutional Review Board to not represent human subjects research, and was therefore exempted from review by both Institutional Review Boards.

### 2.2. Participants

Dentists and physicians with expertise in the treatment of traumatic CMF injuries were recruited from 10 international institutions to participate in expert concept elicitation interviews. Potentially eligible experts were identified through a review of the AO Foundation AOCMF clinical division membership database. Experts were purposively selected to ensure adequate geographical and surgical specialty representation.

Patients who sustained a traumatic CMF injury were identified via referrals from Northwestern University and the University of Texas Health Science Center at San Antonio. Eligible patients were 18 years of age or older, had previously sustained a traumatic CMF injury, were fluent in English, and were able to participate in a verbal interview. Patients were screened for eligibility by a member of the clinic staff and offered the opportunity to participate in an individual interview. The study coordinator explained the study to interested patients. Due to the determination by the Northwestern University Institutional Review Board and the University of Texas Health Science Center at San Antonio Institutional Review Board that the study did not represent human subjects research, informed consent was not required. Patients participated in a telephone or face-to-face interview conducted by a trained researcher. A targeted sampling strategy was employed in an effort to ensure the sample was representative of the full spectrum of experience of traumatic CMF injuries during both the concept elicitation and cognitive debriefing interview phases. Patients received USD 50 compensation for their time.

### 2.3. Expert Concept Elicitation Interviews

Experts participated in a 45–60 min telephone interview conducted by a trained researcher. A semi-structured interview guide was used to elicit experts’ input regarding the symptoms most important to assess following a traumatic CMF injury, as well as their perception of the future applications of this PRO battery.

### 2.4. Patient Concept Elicitation Interviews

Patients participated in either a face-to-face or telephone interview conducted by a trained researcher. Interviews lasted approximately 30 to 60 min. A semi-structured interview guide was used to elicit patients’ input on the most important symptoms to assess post-CMF injury.

### 2.5. Conceptual Model Development and Symptom Battery Development

Following qualitative analysis of expert and patient concept elicitation interviews, the study team identified symptom domains of greatest importance to include in the conceptual model. A conceptual model clearly identifies and categorizes the concepts and symptoms most important to patients and clinicians and how they should be measured. A conceptual model represents the goals of treatment as the “concepts” important in a specific condition and treatment context, with a clear description of outcomes to evaluate when assessing treatment benefit. The development of a conceptual model addresses the question, “What will we be measuring and how will it be organized?” The conceptual model was then used as a structure to inform the development of the symptom battery. Each symptom domain in the conceptual model was further divided into symptom sub-domains to reflect the specific categories of symptoms reported in the interviews. Sub-domain frequency and importance were assessed to guide decisions about item selection. The study team reviewed existing validated PRO instruments and measurement systems, including PROMIS^®®^ [14], Neuro-QoL [15], and the Functional Assessment of Chronic Illness Therapy Measurement System (FACIT) [16] (see Table 1 for a description of these systems), to identify existing measures/items appropriate for the AO CMF Injury Symptom Battery. The study team also revised existing items and drafted new items to adequately measure the concepts identified from the literature review and concept elicitation interviews to include in the symptom battery. When possible, the terminology used by patients during interviews was incorporated into the drafting of new items. Standardized item writing guidelines were followed, including the use of simple and clear language, avoidance of language asking about multiple symptoms in a single item, avoidance of negatively worded questions, and avoidance of culturally biased or colloquial language [17]. The response scale options were retained from existing items and applied to newly created items.

### 2.6. Translatability Review

Newly drafted items were reviewed by a translation expert for translatability, readability, and literacy level. The research team then reviewed the findings from the translatability review and revised, retained, or rejected items for future inclusion based on the translatability review.

### 2.7. Cognitive Debriefing Interviews

Patients participated in 60 min cognitive debriefing interviews via telephone with trained researchers. Participants were first asked to complete a subset of newly drafted items. They were then asked a series of semi-structured questions about their experience responding to the items. The purpose of the cognitive debriefing interviews was to ensure that item content, response scales, and instructions were understood as intended. The research team then reviewed the findings from the cognitive debriefing interviews and revised items based on misinterpretation, confusing syntax, problematic vocabulary, misunderstood response options, and differential meaning across participants to create the AO CMF Injury Symptom Battery.

### 2.8. Data Analysis

Expert and patient interviews were audio recorded and transcribed by a professional medical transcription service. Trained members of the research team used selective qualitative analysis methods and an iterative coding process to identify common themes, create symptom definitions, and develop coding rules to apply to interviewees’ comments [18]. A subset of transcripts was coded independently by two members of the research team. These were then reviewed for consistency and discrepancies were resolved with discussion. The coded comments were then compiled and summarized in frequency tables denoting the number of times certain responses were named. Both frequency and participants’ description of the importance of the symptom were used to develop the conceptual model of the most important CMF symptom domains perceived by experts and patients.

To ensure the representativeness of qualitative findings, research must incorporate key characteristics of the patient population, confirm that the concepts are sufficiently described by study participants, and enroll participants into the study until no new themes are obtained. Theoretical saturation is the point of redundancy when no new themes emerge [19]. Previous research indicates that saturation often occurs within the first 12 interviews and initial themes are sometimes present as early as six interviews [20]. For this study, saturation was considered to have been reached when no new concepts emerged over three consecutive interviews.

## 3. Results

### 3.1. Participant Characteristics

In total, 10 experts and 21 patients participated in concept elicitation interviews. Eight patients participated in cognitive debriefing interviews. Table 2, Table 3 and Table 4 display the sociodemographic and clinical information of participants.

### 3.2. Conceptual Model

**Figure 2:**
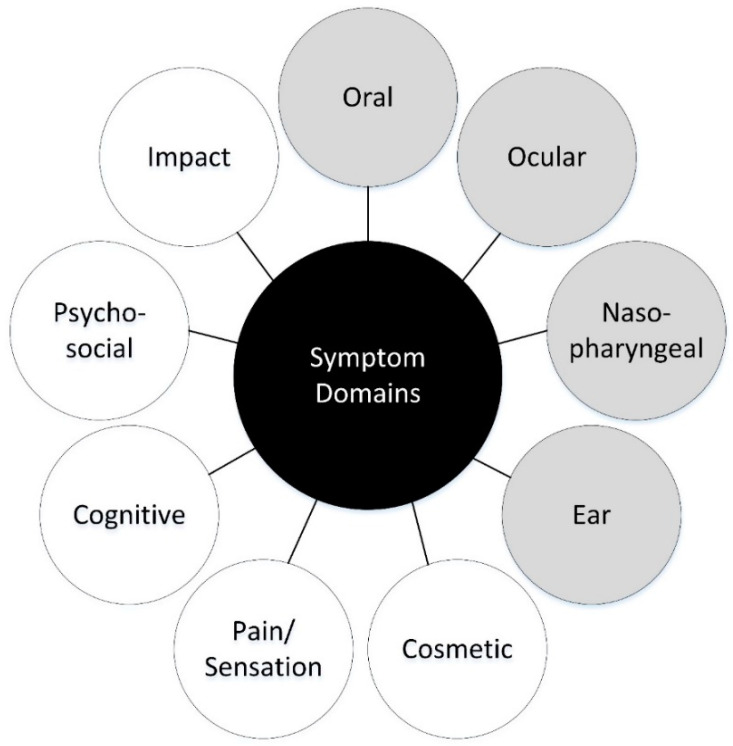
AO CMF Injury Symptom Battery conceptual model.

Nine key symptom domains emerged across the expert and patient interviews. Figure 2 displays the symptom themes. The following four symptom domains were specific to the location of the injury/injuries: oral, ocular, nasopharyngeal, and ear (Table 5). Overall, clinicians identified more symptoms than patients. All site-specific symptoms identified by patients were also identified by clinicians. The following five symptom domains were applicable regardless of the site of the injury: cosmetic, pain/sensation, cognitive, psychosocial, and impact of injury (Table 6). Again, there was consensus between patient- and clinician-identified symptoms. All the symptoms patients mentioned, apart from fatigue, were also noted by clinicians. Guided by the conceptual model, the study team selected individual items to represent each sub-domain from a combination of existing validated items (n = 14) and newly constructed items (n = 38).

### 3.3. Translatability Review Results

**Figure 3:**
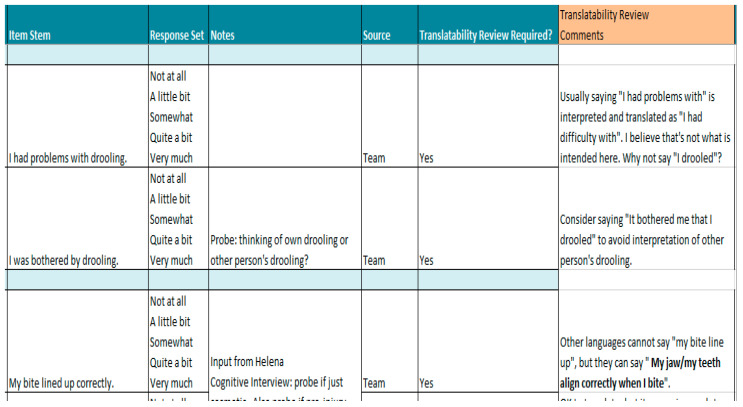
Example of modifications to candidate items following translatability review.

Newly developed and modified existing items underwent a translatability review. Unchanged existing items did not undergo a translatability review because they had been previously reviewed for translatability. Figure 3 provides a brief, non-exhaustive example of modifications to candidate items after a translatability review.

### 3.4. Cognitive Debriefing Results

**Figure 4:**
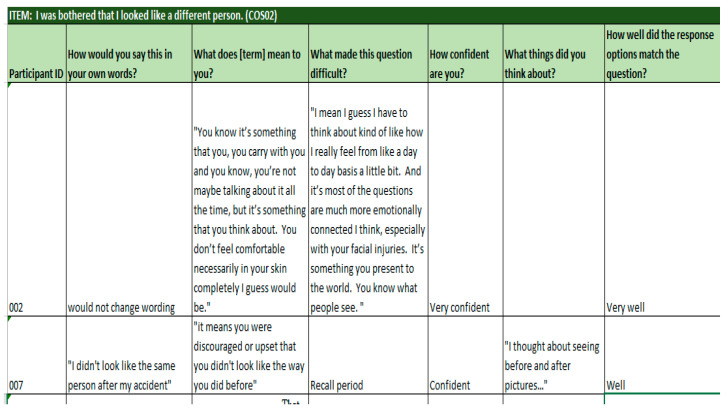
Example of cognitive debriefing findings.

Following a translatability review, cognitive debriefing interviews included newly developed and modified existing items. As with the translatability review, existing items were not included because they had previously been assessed in cognitive debriefing interviews. The findings from cognitive debriefing resulted in the revision of item wording and selection based on participants’ understandability, wording suggestions, and preference decisions between similar items. A notable theme from these interviews involved participants’ preference for items that asked about experiencing a symptom versus being bothered by a symptom. Figure 4 depicts a non-exhaustive example of cognitive debriefing results.

### 3.5. Structure of AO CMF Injury Symptom Battery

Given the resulting four injury site-specific domains and five universal domains that emerged from the battery development process, the AO CMF Injury Symptom Battery was substantively structured as a modular battery in which patients only complete those site-specific modules relevant to their injury site, as well as all universal symptom modules. All respondents are asked screening questions for each site-specific scale to determine if it should be administered (e.g., “Did your face or head injury affect your ear?”). The recall period for all items is the past 7 days, which is consistent with the recall in the majority of PROMIS^®®^ [14], Neuro-QoL [15], and FACIT [16] items. The rationale for this recall period includes avoiding one recall period overlapping with another in longitudinal assessments, minimizing the need for respondents to utilize mental calculations to figure out potentially variable symptoms over a longer period of time, and increasing the likelihood that respondents will be thinking about the “present” when completing the items. The 7-day recall period also allows for the assessment of both acute and chronic symptoms (or those that may vary over time) based upon the selected study design or clinical assessment methodology (single assessment vs. longitudinal). Table 7 depicts the number of items included in each domain. Supplementary Materials displays the AO CMF Injury Symptom Battery (for inquiries related to the scoring of the AO CMF Injury Symptom Battery, please contact clinicalresearch@aofoundation.org).

## 4. Discussion

The assessment of patient-reported outcomes in CMF has been challenging due to the heterogeneous nature of patients’ symptoms depending upon the location and severity of the injury. This suggests that the best measurement approach is a battery in which there is an individual score produced per module, which allows for specificity in outcome assessment in clinical research and practice. Thus, in comparison to other PROs that have been reported to assess CMF injury outcomes [13], the AO CMF Symptom Injury Battery was specifically developed as a condition-specific PRO as opposed to a generic PRO. In the development of the AO CMF Injury Symptom Battery, patients and clinicians identified multiple symptoms associated with injuries to specific parts of the face (e.g., drooling and malocclusion associated with oral injuries). This resulted in the decision to use the mean value of all items for each module of the battery separately (e.g., ocular score, oral score, pain score, etc.) in order to capture both the number of symptoms a patient has, as well as the severity of that symptom. Future research is planned to examine the psychometric properties of this modular approach, although due to the separate scoring for each module, it is expected to function psychometrically, similarly as when multiple measures assessing different domains would be combined in an assessment (e.g., the selection of multiple short forms from the PROMIS^®®^ system which would be scored individually to reflect a unique score for each concept).

Although all but one symptom identified by patients was also identified by clinicians, patients placed greater emphasis on psychosocial outcomes compared to experts. This is consistent with past research highlighting psychosocial distress and trauma following CMF trauma [13,21]. Conversely, experts tended to prioritize more symptoms specific to the site of injury compared to patients, although this may reflect the fact that patients were reporting on their individual symptom experience, whereas experts were describing the full range of symptoms they observe in clinical practice.

Additionally, although experts tended to focus on symptoms that would be expected to resolve following treatment, patients also highlighted various impacts of their injury that were unrelated to CMF injury treatment, such as coping with the trauma that caused the injury and worrying about the long-term consequences of the injury, such as potential changes in cognitive function. This finding underscores the importance of longitudinally implementing PROs in the care of individuals with traumatic CMF injuries given that PROs provide the opportunity for patients to express their unique experience, allowing for more patient-centered care, particularly as it pertains to mental health concerns [21,22].

Physical appearance represented another area in which experts and patients differed in their discussion of symptoms. Specifically, patients described experiencing difficulty adjusting to “looking different” than they looked prior to their injury, even in cases in which there were no longer visible scars, asymmetry, or functional deficits. They frequently spoke of this in the context of the role of the face in their identity, and the way in which looking different than they were used to conferred psychosocial consequences. This is consistent with past research suggesting that individuals who have sustained facial trauma have substantial concerns about their appearance post-injury, which is associated with psychological distress [23], and may experience external stigma leading to social withdrawal [24]. On the contrary, experts were more likely to describe appearance outcomes favorably if there were no visible scars, asymmetry, or functional deficits, and did not reference changes in patients’ appearance compared to pre-injury. This interesting finding suggests that clinicians and patients may have different expectations for treatment outcomes and highlights an opportunity for improved communication about expectations for post-treatment appearance.

Patients also placed greater emphasis on their emotional response to their symptoms compared to experts, who tended to focus more on the resolution of the symptom in response to intervention. For example, patients often described not only the symptom itself but also feeling upset, annoyed, or angry about the symptom. This likely reflects the fact that patients were speaking about their personal CMF injury experience and the difficult nature of separating the experience of a symptom from their emotional response to it, whereas experts focused more on treatable symptoms. This pattern of the findings reinforces the potential value of incorporating PROs into traumatic CMF injury clinical care and research.

The unique battery structure of the AO CMF Injury Symptom Battery enables customized use to assess symptom experience and treatment response over time in the clinical setting. The battery format allows for the selection of domains specific to the nature of injuries, with scores for each domain that can provide useful information clinically to evaluate a variety of quality of life domains. The possible options for the implementation of administration clinically include traditional paper/pencil assessments in clinic, the use of internet connected devices (e.g., tables, smartphones) in clinic, and the integration of the battery into online platforms able to link to the electronic health record. The findings could be used clinically for symptom monitoring, quality improvement, and the identification of instances where patients may benefit from referrals to assist in the treatment of symptoms. The literature has previously highlighted an ongoing follow-up of potentially longer-term consequences of CMF injuries (e.g., psychosocial, appearance, cognitive) to facilitate referrals to other disciplines that may not typically be part of CMF surgical clinics to ensure that ongoing concerns related to the injury itself and the circumstances surrounding it are adequately addressed in addition to the physical symptoms of the injury [21]. The timing of administration of the AO CMF Injury Battery is flexible depending upon the clinical research and practice needs and questions to be addressed. Potential challenges to implementation, which have also been reported elsewhere, include limited knowledge and/or experience with PROs, cost, impact on clinic workflow, concern about the substitution of PROs for traditional clinical outcome measures, and patient burden [6]; however, acceptability for the use of PROs clinically may also relate to efficiency in identifying potential problems, increasing use of technology for administration, satisfaction of regulatory/institution requirements, assistance with patient–provider communication, and decision-making. Future work examining patients’ and providers’ experience and satisfaction with the use of the AO CMF Injury Symptom Battery in clinical practice may be beneficial in identifying barriers to implementation and strategies to overcome them.

The development of the AO CMF Injury Symptom Battery should be considered in light of several limitations. First, although patients were recruited in an effort to enroll patients with a diversity of CMF injuries, the heterogeneous nature of CMF injuries and severities across patients posed some challenges to enrolling a sample that was fully representative of the full CMF injury experience. For example, although experts identified symptoms resulting from injury to the ears as an important area for outcome assessment, none of the patients who participated in the interviews sustained injuries that affected their ear. Similarly, the inclusion criteria only specified that individuals who had sustained a CMF injury were eligible, thus posing a possible limitation since injury severity and time since injury were not included in determining eligibility for participation. Although two different recruitment sources were used in an effort to enhance the sociodemographic diversity of patients, the sample was predominately White and male, which may limit the generalizability of the findings. Given the experts’ discussion of the potential impact of sociodemographic factors on the perceived importance of symptoms (e.g., gender or socioeconomic status differences in the importance of facial appearance), future research to refine the AO CMF Injury Symptom Battery should include a diverse sample of patients in order to enhance the generalizability.

Although the AO CMF Injury Symptom Battery demonstrates preliminary promise as an option to measure patient-reported symptoms, it will benefit from additional refinement in future research. Several future steps are planned to further develop and establish the psychometric properties of this battery. Research is currently underway to examine the validity and reliability of the battery in a geographically diverse sample of patients in the U.S. The development of the battery using input from an English-speaking sample of patients also poses a limitation. Future research should also explore the content validity of the battery with an international sample of participants to ensure it is appropriate for use outside of the U.S. Although items underwent a translatability review to ensure they were written using text and concepts conducive to translation, currently the battery has not yet been translated into other languages. Future efforts should also be directed to the translation of the battery into other languages and the examination of the reliability and validity of translated versions of the battery. Investigations related to establishing clinically meaningful change thresholds also constitute an important next step, which will help to inform the meaningful incorporation of the battery into clinical practice.

## 5. Conclusions

As the first patient-reported outcome battery developed specifically for and with the input of patients who have sustained traumatic CMF injuries, the AO CMF Injury Symptom Battery provides promise as a tool for use in clinical research and practice to measure the most important symptoms in CMF injuries. The assessment of patient-reported outcomes in this patient population enables improved assessment of aspects of the CMF injury experience that only patients can report on. It could be used as a useful patient-reported outcome tool alongside other more generic (non-condition-specific) measures of quality of life and functional outcomes, both in research and clinical practice, particularly given that incidents/accidents resulting in CMF injuries commonly include other non-CMF-related injuries, with the potential to negatively affect physical function and overall quality of life. Its incorporation into clinical research also provides the opportunity to examine long-term outcomes not always captured by the short-term clinical follow-up. It may also serve as a useful resource to identify symptoms that might warrant clinical referrals to other disciplines (e.g., mental health, pain management). Future research will address the psychometric properties of the battery that were beyond the scope of the initial development process.

## Figures and Tables

**Table 1:** Existing validated measurement systems reviewed during item selection.

Measurement System	Description
PROMIS^®®^ [14]	Over 300 measures of physical, mental, and social health across general population and all health conditions Developed and evaluated using rigorous measurement and psychometric methods Translations in Spanish and other languages available
Neuro-QoL [15]	Brief, valid, and reliable measures assessing physical, mental, and social experiences of individuals with neurological conditions Translations in Spanish and other languages available
FACIT [16]	Over 700 items and 100 validated measures to assess the management of chronic illness in multiple diseases, treatments, and conditions Translations in more than 80 languages available Estimated administration time for any one FACIT assessment ≤ 15 min

**Table 2:** Concept elicitation expert characteristics (N = 10).

Characteristic		N (%)
Gender		
	Male	10 (100)
Location		
	Austria	1 (10)
	Brazil	1 (10)
	Egypt	1 (10)
	Finland	1 (10)
	Germany	1 (10)
	Mexico	1 (10)
	Singapore	1 (10)
	United States	3 (30)
Surgical Specialty		
	Craniomaxillofacial	4 (40)
	Neurosurgery	1 (10)
	Plastic/reconstructive	4 (40)
	Oculoplastic/Ophthalmology	1 (10)
Median Years in Practice (Range)		26.5 (13–43)
Median Years Treating CMF Injury Patients (Range)		25 (15–41)

**Table 3:** Concept elicitation patient sociodemographic and injury characteristics (N = 21).

Characteristic	Number of Participants (%)
Age mean	32.43
Age range	18–69
Gender	
Female	4 (19.05)
Male	17 (80.95)
Race	
Asian	2 (9.52)
Black or African American	1 (4.76)
Other	2 (9.52)
White	16 (76.19)
Ethnicity	
Hispanic	1 (4.76)
Non-Hispanic	20 (95.24)
Injury site	
Eyebrow to supraorbital ridge	1 (4.76)
Face	3 (16.67)
Face and mandible	1 (4.76)
Lip to chin	1 (4.76)
Mandible	4 (19.05)
Nasal	6 (28.57)
Nasal and orbital	1 (4.76)
Nasolabial fold	1 (4.76)
Zygoma	3 (16.67)
Injury type	
Fracture	18 (85.71)
Gunshot wound	1 (4.76)
Laceration	2 (9.52)
Accident	14 (66.67)
Automobile	1 (4.76)
Fall	8 (38.10)
Motorcycle	1 (4.76)
Sailing	1 (4.76)
Sports	3 (16.67)
Violence	7 (33.33)
Time since injury—mean (months)	10.62

**Table 4:** Cognitive debriefing interview patient sociodemographic and injury characteristics (N = 8).

Characteristic	Number of Participants (%)
Age mean	31.43
Age range	18–65
Gender	
Female	1 (12.50)
Male	7 (87.50)
Race	
Black or African American	1 (12.50)
Other	2 (25.00)
White	5 (62.50)
Ethnicity	
Hispanic	1 (12.50)
Non-Hispanic	7 (87.50)
Injury site ^a^	
Facial	4 (50.00)
Mandible	2 (25.00)
Nasal	4 (50.00)
Orbital	2 (25.00)
Zygoma	2 (25.00)
Injury type	8
Fracture	8 (100.00)
Accident	4 (50.00)
Automobile	1 (12.50)
Fall	2 (25.00)
Sports	1 (12.50)
Violence	4 (50.00)

^a^ Several patients had multiple injury sites so N will not add up to 8 for this section of the table since several patients fell into multiple categories.

**Table 5:** Comparison of patient and expert endorsement of important injury site-specific sub-domains.

Symptom Domain		% of Patients Who Endorsed	% of Experts Who Endorsed	Resulting Items Included in Battery
Oral Domain ^a^				
	Drooling	00.0	30.0	I drooled when awake.
	Malocclusion	30.0	80.0	My jaw aligns correctly when I bite.
	Taste sensitivity problems	10.0	20.0	I had a problem with my sense of taste.
	Jaw problems	70.0	80.0	I had problems with my jaw.
	Tooth problems	10.0	40.0	I had problems with a tooth.
	Swallowing problems	00.0	30.0	I had trouble swallowing.
	Lip problems	00.0	30.0	I had problems with my lips.
	Tongue problems	00.0	30.0	I had problems with my tongue.
Ocular Domain ^b^				
	Vision problems	33.3	70.0	I had problems with my vision.
	Double vision	16.7	90.0	I had double vision.
	Eyelash problems	00.0	10.0	My eyelashes irritated my eye(s).
	Tearing problems	16.7	30.0	My eyes produced tears normally.
	Eyelid problems	33.3	80.0	I had problems opening or closing my eye(s).
	Eyeball problems	8.3	10.0	My eye was itchy.
	Light sensitivity	33.3	30.0	My eyes were sensitive to light.
Nasopharyngeal Domain ^c^				
	Breathing problems	50.0	80.0	I had difficulty breathing through my nose.
	Sinus problems	8.3	40.0	I had sinus problems.
	Sense of smell problems	8.3	50.0	I had problems with my sense of smell.
Ear Domain ^d^				
	Deafness	00.0	10.0	I had trouble hearing.
	Hearing problems	00.0	30.0	I heard unusual noises (e.g., ringing, clicking).

^a^ Patients with oral injuries, n = 10. ^b^ Patients with ocular injuries, n = 12. ^c^ Patients with nasopharyngeal injuries, n = 12. ^d^ Patients with ear injuries, n = 0.

**Table 6:** Comparison of patient and expert endorsement of important injury universal sub-domains.

Symptom Domain		% of Patients Who Endorsed (N = 21)	% of Experts Who Endorsed (N = 10)	Resulting Items Included in Battery
Cosmetic Domain				
	Facial appearance problems	42.9	50.0	My face looked different because of my injury.
	Scarring	28.6	50.0	I had a scar on my face or scalp.
	Palsy	4.8	40.0	I had trouble moving parts of my face.
Pain/Sensation Domain				
	Localized pain/discomfort	76.2	90.0	I had pain in my face or head.
				I had tenderness in my face or scalp.
	Headache pain	42.9	30.0	I had headaches.
				I had migraines.
	Temperature sensitivity	23.8	20.0	
	Numbness	52.4	70.0	I had numbness in my face or scalp.
	Abnormal sensations	28.6	20.0	I had unusual sensations in my face or scalp.
	Vertigo	9.5	10.0	I felt dizzy.
Cognitive Domain				
	Cognitive functioning defects	14.3	40.0	My thinking has been slow.
				It has seemed like my brain was not working as well as usual.
				I have to work harder than usual to keep track of what I was doing.
				I have had trouble shifting back and forth between different activities that require thinking.
Psychosocial Domain				
	Body image problems	52.4	30.0	I felt self-conscious about my appearance.
				I was bothered by changes in my appearance because of my injury.
	Depression	23.8	60.0	I felt depressed.
	Anxiety	61.9	20.0	I was concerned about the long-term consequences of my injury.
				I felt anxious.
				I paid very close attention to my surroundings.
	Medication problems	14.3	20.0	I had trouble managing my pain medication.
	Relationship problems	19.0	50.0	My relationships have been strained due to my injury.
				I felt like a burden to others due to my injury.
	Social problems	42.9	60.0	I am satisfied with my current level of social activity.
	Adjustment problems	28.6	20.0	I had trouble coping with the consequences of my injury.
	Psychosocial change not otherwise specified	52.4	50.0	I felt guilty about my injury.
Impact Domain				
	Basic activities of daily living	76.2	80.0	I am able to eat normally.
				I can take care of myself (e.g., bathing, dressing)
	Instrumental activities of daily living	71.4	90.0	I have trouble taking care of my regular personal responsibilities.
				I am satisfied with my ability to do leisure activities.
				I am able to keep up with my work responsibilities (including work at home).
	Communication	47.6	70.0	I was able to communicate with others.
	Fatigue	23.8	00.0	I felt fatigued.
	Impact not otherwise specified	23.8	10.00	I had a problem with my sleep.

**Table 7:** AO CMF Injury Symptom Battery item numbers per domain.

Domain	Number of Items	Estimated Completion Time, Minutes
Ocular	7	1.2
Oral	8	1.3
Nasopharyngeal	3	0.5
Ear	2	0.3
Pain/Sensation	7	1.2
Cognitive	4	0.7
Cosmetic	3	0.5
Psychosocial	12	2.0
Impact	8	1.3

Note. Previous calculations indicate that most individuals take approximately 10 s to complete an item online (approximately 5–6 items per minute).

## Data Availability

The original contributions presented in this study are included in the article/Supplementary Material. Further inquiries can be directed to the corresponding author.

## Supplementary Materials

The following supporting information can be downloaded at: https://www.mdpi.com/article/10.3390/jcm13237156/s1, AO CMF Injury Symptom Battery.

## Abbreviations

AO	AO Foundation
CMF	Craniomaxillofacial
PRO	Patient-reported outcome
