# Supplementary material for: Measuring Patient-Reported Outcomes Following Traumatic Craniomaxillofacial Injuries: Development of the AO CMF Injury Symptom Battery
Source: J Clin Med. 2024 Nov 26;13(23):7156. doi: 10.3390/jcm13237156 (PMC11641857; doi:10.3390/jcm13237156)
Supplement: Supplementary file 1 [file jcm-13-07156-s001.zip › jcm-3315862-supplementary.pdf]

AO CMF

Eye Questions

Please answer the following questions about how you felt in the past 7 days related to your face or head injury.

		Yes	No
**1** OCUL00	Did your face or head injury affect your eye or eyes?	☐ 1	☐ 0
		Yes→ Go to question OCUL01.<br>No→ Skip to #9 on Page 2.	

	**In the past 7 days…**	**Not at all**	**A little bit**	**Somewha t**	**Quite a bit**	**Very much**
**2** OCUL01	I had problems with my vision …………….	☐ 1	☐ 2	☐ 3	☐ 4	☐ 5
**3** OCUL05	I had double vision…………………………	☐ 1	☐ 2	☐ 3	☐ 4	☐ 5
**4** OCUL13	My eyelashes irritated my eye(s)…………...	☐ 1	☐ 2	☐ 3	☐ 4	☐ 5
**5** OCUL25	I had problems opening or closing my eye(s)……………………………………	☐ 1	☐ 2	☐ 3	☐ 4	☐ 5
**6** OCUL27	My eye was itchy…………………………..	☐ 1	☐ 2	☐ 3	☐ 4	☐ 5
**7** OCUL30	My eyes were sensitive to light…………….	☐ 1	☐ 2	☐ 3	☐ 4	☐ 5
**8** OCUL32	My eyes produced tears normally…………..	☐ 5	☐ 4	☐ 3	☐ 2	☐ 1

The “AO CMF Injury Symptom Battery” is owned and copyrighted by AOCMF
Use of the “AO CMF Injury Symptom Battery” requires written permission from AOCMF.

AO CMF

Mouth and Jaw Questions

Please answer the following questions about how you felt in the past 7 days related to your face or head injury.

		Yes	No
9 ORAL00	Did your face or head injury affect your mouth or jaw?	☐ 1	☐ 0
		Yes→ Go to question ORAL01. No→ Skip to #18 on Page 3.	

In the past 7 days…

		Not at all	A little bit	Somewhat	Quite a bit	Very much
10 ORAL01	I drooled when awake……………………….	☐ 1	☐ 2	☐ 3	☐ 4	☐ 5
11 ORAL09	I had a problem with my sense of taste…….	☐ 1	☐ 2	☐ 3	☐ 4	☐ 5
12 ORAL12	I had problems with my jaw………………..	☐ 1	☐ 2	☐ 3	☐ 4	☐ 5
13 ORAL18	I had problems with a tooth………………...	☐ 1	☐ 2	☐ 3	☐ 4	☐ 5
14 ORAL23	I had trouble swallowing…………………...	☐ 1	☐ 2	☐ 3	☐ 4	☐ 5
15 ORAL27	I had problems with my lips………………..	☐ 1	☐ 2	☐ 3	☐ 4	☐ 5
16 ORAL30	I had problems with my tongue…………….	☐ 1	☐ 2	☐ 3	☐ 4	☐ 5
17 ORAL03	My jaw aligns correctly when I bite………..	☐ 5	☐ 4	☐ 3	☐ 2	☐ 1

The "AO CMF Injury Symptom Battery" is owned and copyrighted by AOCMF
Use of the "AO CMF Injury Symptom Battery" requires written permission from AOCMF.

AO CMF

Nose Questions

Please answer the following questions about how you felt in the past 7 days related to your face or head injury.

		Yes	No
18<br>NASAL00	Did your face or head injury affect your nose or sinuses?	☐<br>1	☐<br>0
		Yes→ Go to question NASAL01.<br>No→ Skip to #22 on Page 4.	

In the past 7 days…

		Not at all	A little bit	Somewhat	Quite a bit	Very much
19<br>NASAL01	I had difficulty breathing through my nose………………………………………… .	☐<br>1	☐<br>2	☐<br>3	☐<br>4	☐<br>5
20<br>NASAL03	I had sinus problems………………………	☐<br>1	☐<br>2	☐<br>3	☐<br>4	☐<br>5
21<br>NASAL05	I had problems with my sense of smell……	☐<br>1	☐<br>2	☐<br>3	☐<br>4	☐<br>5

The "AO CMF Injury Symptom Battery" is owned and copyrighted by AOCMF
Use of the "AO CMF Injury Symptom Battery" requires written permission from AOCMF.

AO CMF

Ear Questions

Please answer the following questions about how you felt in the past 7 days related to your face or head injury.

		Yes	No
22 EAR00	Did your face or head injury affect your ear?	☐ 1	☐ 0
		Yes→ Go to question EAR01. No→ Skip to #25 on Page 4.	

In the past 7 days…

		Not at all	A little bit	Somewhat	Quite a bit	Very much
23 EAR01	I had trouble hearing…………………………	☐ 1	☐ 2	☐ 3	☐ 4	☐ 5
24 EAR03	I heard unusual noises (e.g., ringing, clicking)…………………………………….. .	☐ 1	☐ 2	☐ 3	☐ 4	☐ 5

The “AO CMF Injury Symptom Battery” is owned and copyrighted by AOCMF
Use of the “AO CMF Injury Symptom Battery” requires written permission from AOCMF.

AO CMF

Pain and Sensation Questions

Please answer the following questions about how you felt in the past 7 days related to your face or head injury.

In the past 7 days…

		Not at all	A little bit	Somewhat	Quite a bit	Very much
25 PAINS01	I had pain in my face or head………………	☐ 1	☐ 2	☐ 3	☐ 4	☐ 5
26 PAINS05	I had tenderness in my face or scalp………..	☐ 1	☐ 2	☐ 3	☐ 4	☐ 5
27 PAINS18	I had numbness in my face or scalp………..	☐ 1	☐ 2	☐ 3	☐ 4	☐ 5

		Never	Rarely	Sometimes	Often	Always
28 PAINS14	I had headaches……………………………..	☐ 1	☐ 2	☐ 3	☐ 4	☐ 5
29 PAINS15	I had migraines……………………………..	☐ 1	☐ 2	☐ 3	☐ 4	☐ 5
30 PAINS23	I felt dizzy…………………………………..	☐ 1	☐ 2	☐ 3	☐ 4	☐ 5
31 PAINS17	I had unusual sensations in my face or scalp……………………………………….. .	☐ 1	☐ 2	☐ 3	☐ 4	☐ 5

The "AO CMF Injury Symptom Battery" is owned and copyrighted by AOCMF
Use of the "AO CMF Injury Symptom Battery" requires written permission from AOCMF.

AO CMF

Cognitive Questions

Please answer the following questions about how you felt in the past 7 days related to your face or head injury.

	**In the past 7 days…**	**Never**	**Rarely (once)**	**Sometimes (two or three times)**	**Often (about once a day)**	**Very often (several times a day)**
32 PC2rCMF	My thinking has been slow………………	☐ 1	☐ 2	☐ 3	☐ 4	☐ 5
33 PC35rCMF	It has seemed like my brain was not working as well as usual…………………	☐ 1	☐ 2	☐ 3	☐ 4	☐ 5
34 PC36rCMF	I have had to work harder than usual to keep track of what I was doing…………..	☐ 1	☐ 2	☐ 3	☐ 4	☐ 5
35 PC42rCMF	I have had trouble shifting back and forth between different activities that require thinking………………………………..	☐ 1	☐ 2	☐ 3	☐ 4	☐ 5

The "AO CMF Injury Symptom Battery" is owned and copyrighted by AOCMF
Use of the "AO CMF Injury Symptom Battery" requires written permission from AOCMF.

AO CMF

Appearance Questions

Please answer the following questions about how you felt in the past 7 days related to your face or head injury.

	**In the past 7 days…**	**Not at all**	**A little bit**	**Somewhat**	**Quite a bit**	**Very much**
36 COS04	I had trouble moving parts of my face……….	☐ 1	☐ 2	☐ 3	☐ 4	☐ 5
37 COS08	My face looked different because of my injury……………………………………… …	☐ 1	☐ 2	☐ 3	☐ 4	☐ 5
38 COS09	I had a scar on my face or scalp……………..	☐ 1	☐ 2	☐ 3	☐ 4	☐ 5

The “AO CMF Injury Symptom Battery” is owned and copyrighted by AOCMF
Use of the “AO CMF Injury Symptom Battery” requires written permission from AOCMF.

AO
CMF

Psychosocial Questions

Please answer the following questions about how you felt in the past 7 days related to your face or head injury.

	**In the past 7 days…**	**Not at all**	**A little bit**	**Somewhat**	**Quite a bit**	**Very much**
39 PSY01	I felt self-conscious about my appearance……………………………….…	☐ 1	☐ 2	☐ 3	☐ 4	☐ 5
40 PSY13	I was bothered by changes in my appearance because of my injury…………..	☐ 1	☐ 2	☐ 3	☐ 4	☐ 5
41 PSY06	I was concerned about the long-term consequences of my injury…………………	☐ 1	☐ 2	☐ 3	☐ 4	☐ 5
42 PSY23	I had trouble managing my pain medication……………………………….…	☐ 1	☐ 2	☐ 3	☐ 4	☐ 5
43 PSY10	My relationships have been strained due to my injury…………………………………...	☐ 1	☐ 2	☐ 3	☐ 4	☐ 5
44 PSY11	I felt like a burden to others due to my injury……………………………………….	☐ 1	☐ 2	☐ 3	☐ 4	☐ 5
45 PSY18	I had trouble coping with the consequences of my injury………………………………....	☐ 1	☐ 2	☐ 3	☐ 4	☐ 5
46 PSY20	I felt guilty about my injury………………..	☐ 1	☐ 2	☐ 3	☐ 4	☐ 5
		**Never**	**Rarely**	**Sometimes**	**Often**	**Always**
47 EDDEP29	I felt depressed……………………………	☐ 1	☐ 2	☐ 3	☐ 4	☐ 5
48 EDANX05	I felt anxious………………………………	☐ 1	☐ 2	☐ 3	☐ 4	☐ 5

The "AO CMF Injury Symptom Battery" is owned and copyrighted by AOCMF
Use of the "AO CMF Injury Symptom Battery" requires written permission from AOCMF.

49<br>PSY08	I paid very close attention to my surroundings…………………………………..	☐<br>1	☐<br>2	☐<br>3	☐<br>4	☐<br>5

	**In the past 7 days…**	**Not at all**	**A little bit**	**Somewhat**	**Quite a bit**	**Very much**
50<br>SRPSAT10r1r	I am satisfied with my current level of social activity………………………………	☐<br>5	☐<br>4	☐<br>3	☐<br>2	☐<br>1

The "AO CMF Injury Symptom Battery" is owned and copyrighted by AOCMF
Use of the "AO CMF Injury Symptom Battery" requires written permission from AOCMF.

AO CMF

Injury Impact

Please answer the following questions about how you felt in the past 7 days related to your face or head injury.

		Never	Rarely	Sometimes	Often	Always
51 SRPPPER31_CaPSr	I have trouble taking care of my regular personal responsibilities…………………	☐ 1	☐ 2	☐ 3	☐ 4	☐ 5
		**Not at all**	**A little bit**	**Somewhat**	**Quite a bit**	**Very much**
52 SRPSAT23r1r	I am satisfied with my ability to do leisure activities………………………………….. .	☐ 5	☐ 4	☐ 3	☐ 2	☐ 1
	**In the past 7 days…**	**Not at all**	**A little bit**	**Somewhat**	**Quite a bit**	**Very much**
53 IMP02	I am able to eat normally…………………	☐ 5	☐ 4	☐ 3	☐ 2	☐ 1
54 IMP03	I can take care of myself (e.g., bathing, dressing)………………………………….. ..	☐ 5	☐ 4	☐ 3	☐ 2	☐ 1
55 HN10r	I was able to communicate with others…………………………………….. ..	☐ 5	☐ 4	☐ 3	☐ 2	☐ 1
		**Never**	**Rarely**	**Sometimes**	**Often**	**Always**
56 NQPRF34cmf	I can keep up with my work responsibilities (including work at home)…...	☐ 5	☐ 4	☐ 3	☐ 2	☐ 1
		**Not at all**	**A little bit**	**Somewhat**	**Quite a bit**	**Very much**
57 SLEEP20	I had a problem with my sleep…………….	☐ 1	☐ 2	☐ 3	☐ 4	☐ 5
58 HI7r	I felt fatigued………………………………	☐ 1	☐ 2	☐ 3	☐ 4	☐ 5

The “AO CMF Injury Symptom Battery” is owned and copyrighted by AOCMF
Use of the “AO CMF Injury Symptom Battery” requires written permission from AOCMF.
